# Determination of Micro-Events and Microcracks in the Compressive Strength of Concrete Using the 3D Acoustic Spectrum in the Low Frequency and Infrasound

**DOI:** 10.3390/ma19153331

**Published:** 2026-08-05

**Authors:** Dominik Logoń

**Affiliations:** Institute of Building Engineering, University of Wroclaw, 50-370 Wroclaw, Poland; dominik.logon@pwr.edu.pl

**Keywords:** acoustic emission AE, 3D acoustic spectrum, cement composites, destruction process, micro-events, micro and macrocracks

## Abstract

Acoustic emission (AE) measurements are commonly used in studies of cementitious composites subjected to various types of loading. Recording macrocracks that result in a decrease in stress is not relatively difficult. This paper focuses on the possibility of identifying micro-events and microcracks during the compression of concrete with dispersed reinforcement. Most AE studies on cement composites correspond to a reduction in stress exceeding the elastic range defined by Hooke’s law, typically associated with the formation of the first crack and detected in the medium- and high-frequency ranges. However, identifying micro-events which do not reduce stress beyond the elastic range is difficult. This study demonstrates that such micro-events can be detected using low-frequency sound and infrasound. In many papers, medium- and high-frequency acoustic signals are effective for recording macrocracks or reinforcement damage. In this work, a 3D acoustic spectrum was used to analyze recorded data in the infrasound range in a concrete compressive test. This approach proved to be the most effective method for determining the critical point f_cr_ (the end of the elastic range) regarding low-intensity micro-events and microcracks. This type of micro-damage has no significant influence on the linear stress–strain correlation at f_cr_. The results indicate that identifying micro-events and low-intensity microcracks using medium- and high-frequency acoustic signals is not possible and that infrasound should be considered for the detection. Significant differences in stress and displacement corresponding to f_cr_ and f_max_ were confirmed in concrete compressive tests. The results indicate that accurately determining f_cr_ is required for correctly assessing the durability of cementitious composites.

## 1. Introduction

Monitoring the destruction process of cementitious composites using acoustic emission has been widely applied for many years [1,2,3]. Tests are conducted under various loading conditions [4]. Most studies focus on tensile and bending testing of mortars and concretes [5,6,7,8,9].

Various measurement techniques have been employed, primarily concentrating on the recording of AE signals in the medium and high-frequency ranges [10,11,12,13,14,15,16]. The identification of macrocracks at maximum load and the monitoring of the failure process have been the subject of numerous studies [17,18,19,20,21,22]. The primary application of this research is related to structural safety assessment and the diagnosis of structural elements [23,24,25,26,27].

Most existing studies focus on the analysis of acoustic effects occurring after the formation of microcracks and macrocracks [28,29,30,31,32,33,34,35]. Different types of damage have been recorded, including the occurrence of micro-events [36,37]. Micro-events comprise various phenomena, such as fiber pull-out, microstructural degradation, and detachment of coarse aggregate from the cement matrix. It was stated that the correlation of these effects with AE signals requires further investigation [38,39,40,41].

Previous studies have indicated a correlation between the acoustic spectrum and individual damage processes and their propagation, such as micro- and macrocracking, reinforcement breakage and pull-out, and the propagation of these effects [37,38]. The 3D acoustic spectrum enables the observation of acoustic effects over a wide frequency range and the correlation of specific frequency ranges with individual damage mechanisms [39,40].

Determining the micro-event signal from the background noise using traditional methods (in the mid- and high-frequency ranges) is not possible. To investigate the deterioration of material properties and their microstructure, combined analytical techniques were proposed [41,42,43,44].

The research indicated that the increase in the EA sum is information about f_cr_ (range of Hooke’s law) [3]. The basic problem concerned the possibility of determining f_cr_ when the intensity of events is low. The studies show a concentration of micro-cracks before the appearance of f_max_, but do not record low-intensity micro-events at the f_cr_, which makes it difficult to correlate with acoustic effects [45]. One of the reasons was the lack of appropriate measurement devices (including an understanding) to search for microevents in the range of infrasound. Various alternative methods of micro-destruction research using EA are proposed [46,47].

In this paper, the 3D acoustic spectrum, mainly in infrasound and low frequency, was tested, indicating the effectiveness of this method for identifying micro-events and microcracks. Accurate determination of the critical stress f_cr_ enables correct assessment of the durability of the cement composites.

## 2. Materials and Methods

The tests were conducted on concrete reinforced with dispersed steel fibers with the following properties (hooked): tensile strength f_t_ = 1350 MPa; diameter d = 0.70 mm; length l = 50 mm; and l/d = 70. The concrete mixture contained 330 kg/m^3^ of Górażdże cement (Poland) CEM II/B-M (S-V) 42.5 N cement with a water/cement ratio (w/c) of 0.48. The natural aggregate (0/16 mm) content was 1820 kg/m^3^.

The displacement rate of the traverse during the compression test was 0.5 MPa/s. The specimen cross section was 150 mm × 150 mm. Six samples were prepared for each fiber content of 10 kg/m^3^, 15 kg/m^3^, and 17 kg/m^3^.

To interpret the acoustic effects, the Spectra PLUS-SC software (Pioneer Hill Software LLC, Sequim, WA, USA, version 5.3.3.5) was used, which enables the visualization of the 3D acoustic spectrum. Due to the large volume of AE data and 3D spectra, it is not feasible to present all results. Therefore, one representative sample from each series was selected to illustrate the physico-mechanical properties and the most significant acoustic effects.

Table 1 presents two types of microphones with different characteristics. The first microphone is designed for the infrasound range covering frequencies from 0.5 Hz up to 20 kHz. The second microphone is less sensitive but offers a wider frequency range from 4 Hz to 70 kHz.

**Figure 1:**
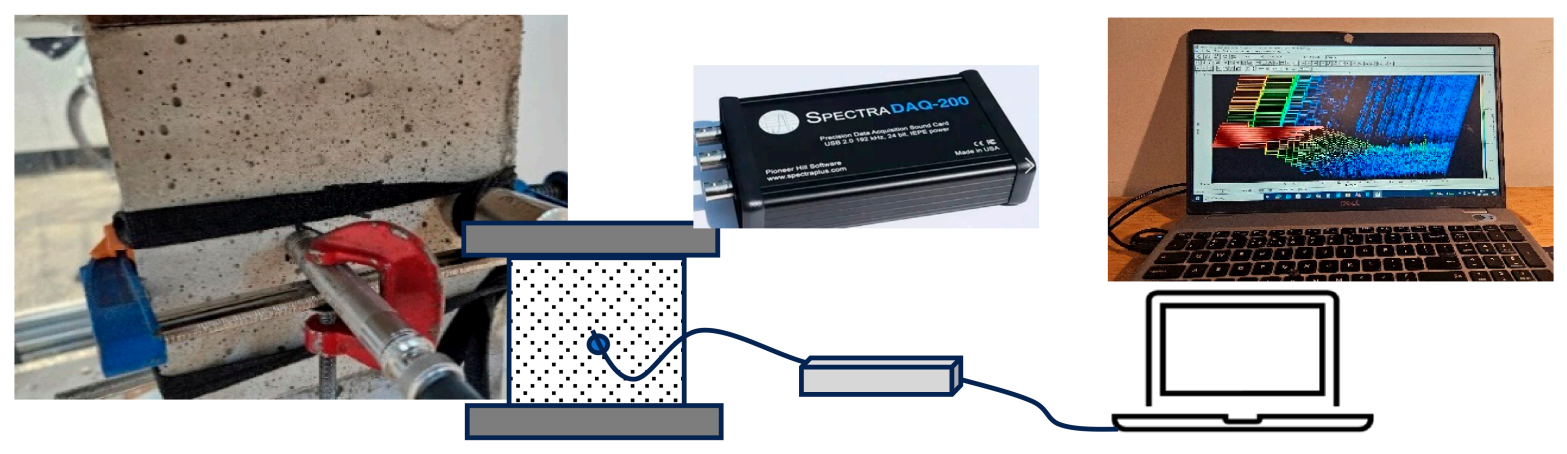
Acoustic effects measurement.

Table 2 presents symbols of concrete with different fiber volume and selected microphone type. Figure 1 illustrates the measurement setup.

**Figure 2:**
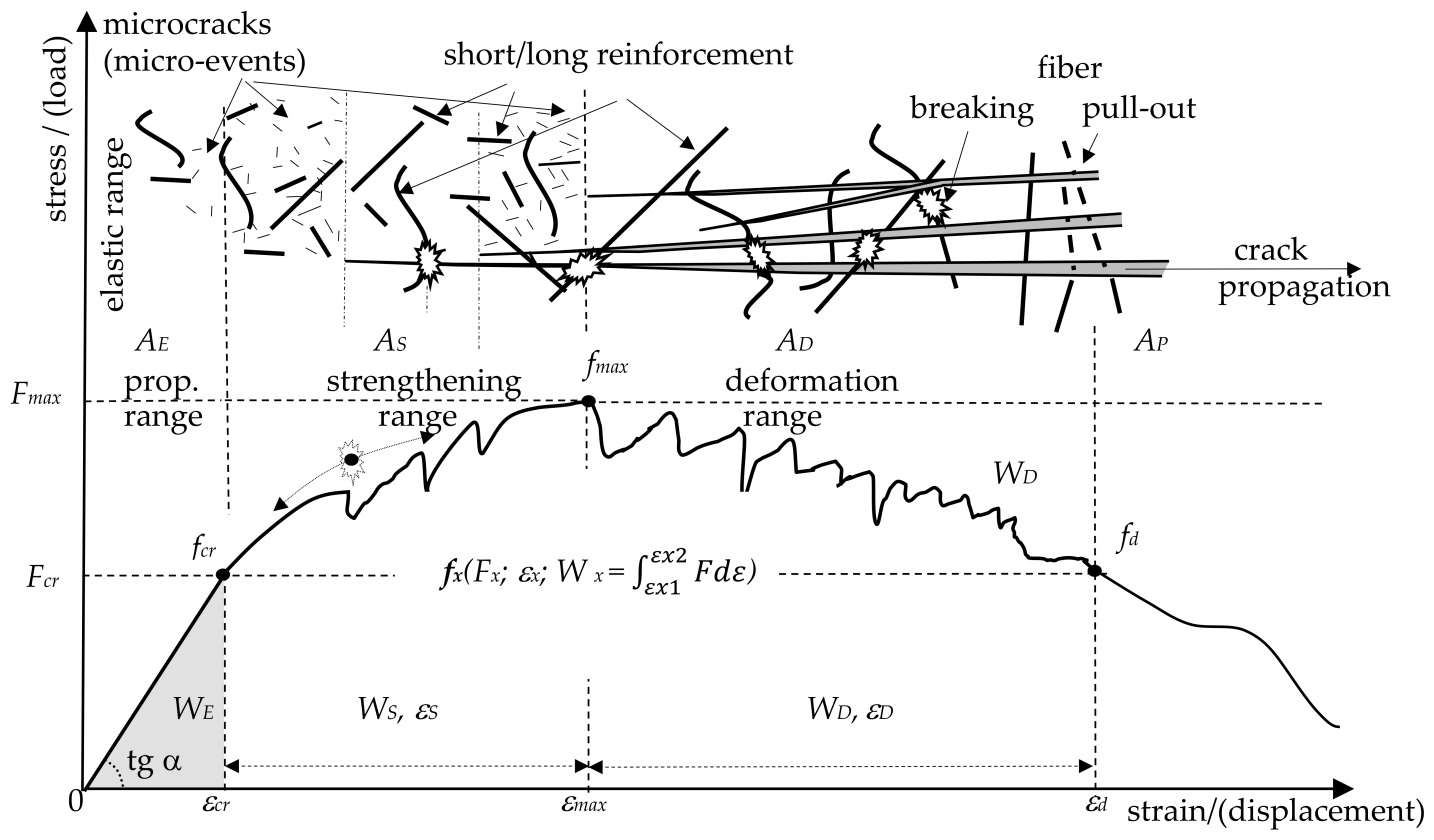
Load–displacement curve of fiber-reinforced cement composites.

Figure 2 shows a diagram of ESD fiber-reinforced cement composites during stress–strain (load/displacement) in the compressive/bending test. The composites exhibit significantly higher stresses and deformations after exceeding the elastic range. For each point f_x_ (compressive/bending strength) on the stress–strain curve, the corresponding force F_x_, strain ε_x_ (displacement d) and absorbed energy W_x_ (calculated as the area under the curve) can be determined.

## 3. Results

**Figure 3:**
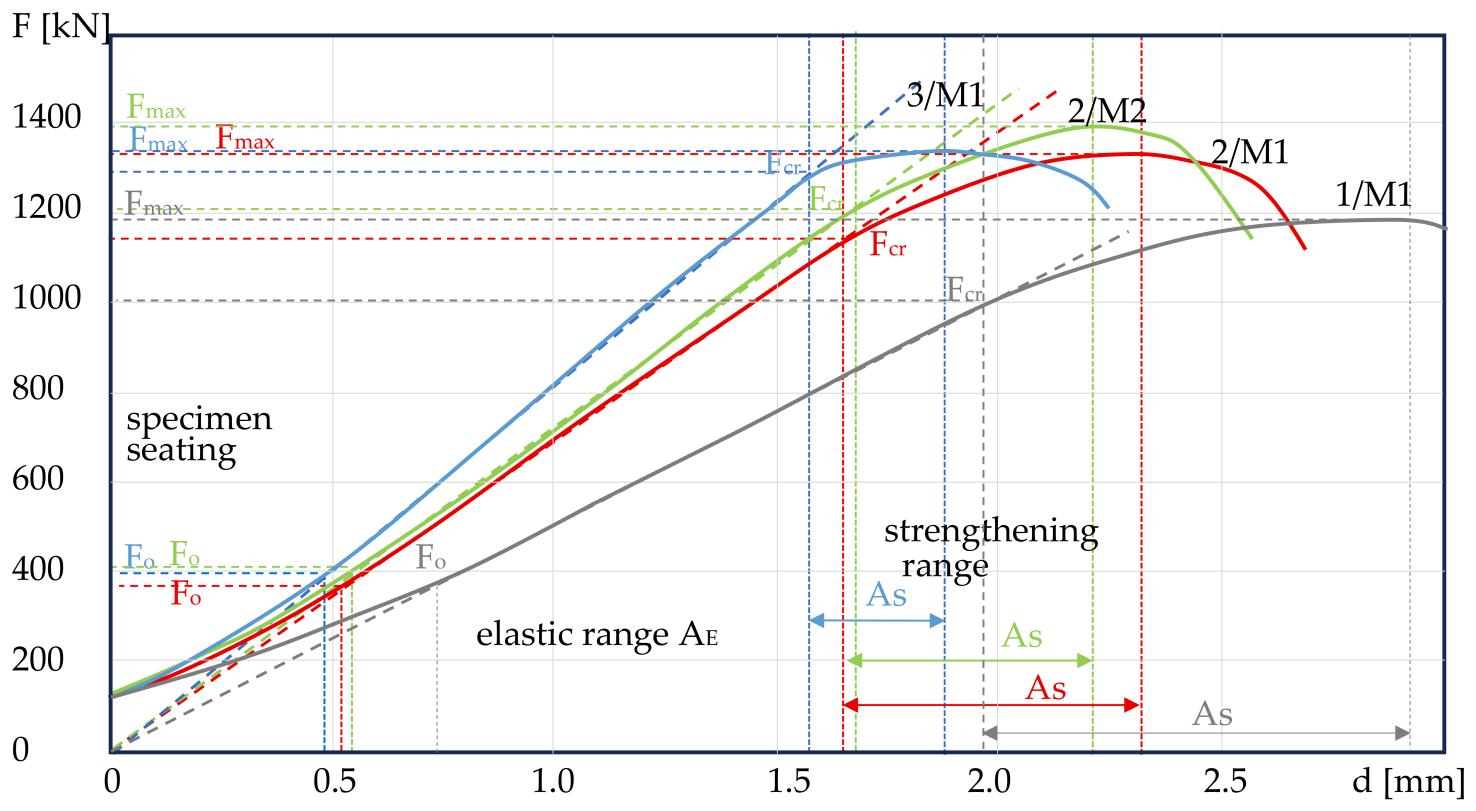
Load–displacement curves of concrete under compressive strength test.

The mechanical properties of the samples under compressive load are presented in Table 3 and Figure 3. The values of force, deflection and stress were determined for the critical point f_cr_ (elastic range) and for f_max_. In addition, the slope of the load–displacement curve (tgα = F_cr_/d_cr_) and absorbed energy W_S_ (the area under the curve between f_cr_ and f_max_) are included. Deflection was measured as traverse displacement, which resulted in a non-linear correlation up to the point F_o_ due to specimen seating. To facilitate comparison of the load–displacement curves, the proportional correlation (the initial portion of the curve F_cr_ up to F_o_) was extrapolated to the origin (due to specimen seating), representing the elastic range, A_E_. The time of the traverse displacement between f_cr_ and f_max_ was marked as t_S_ and the displacement in the strengthening range was marked as d_s_ = d_max_ − d_cr_.

**Figure 4:**
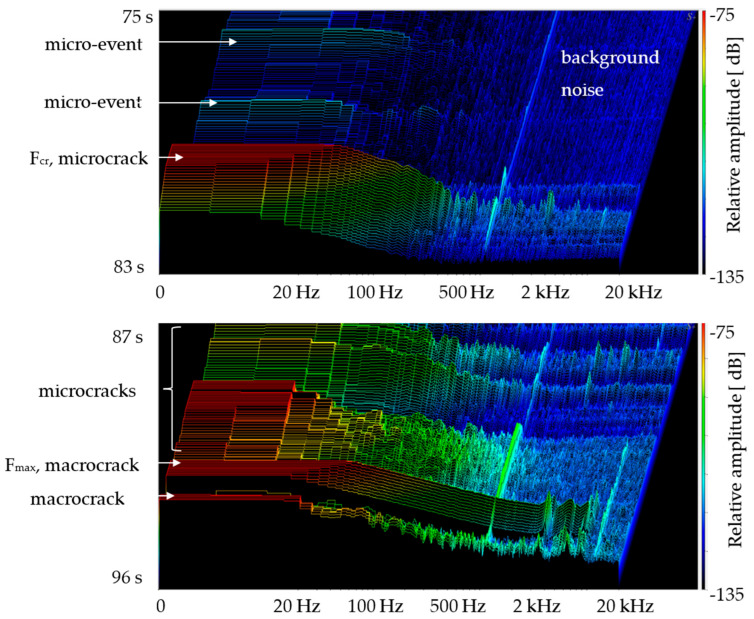
Acoustic spectrum 3D—1/M1 sample during compression test.

**Figure 5:**
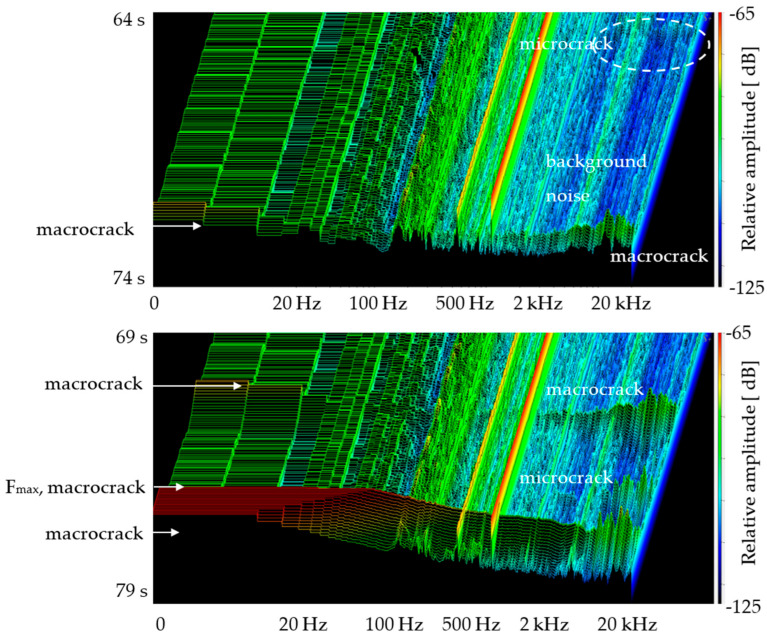
Acoustic spectrum 3D—2/M2 sample during compression test.

**Figure 6:**
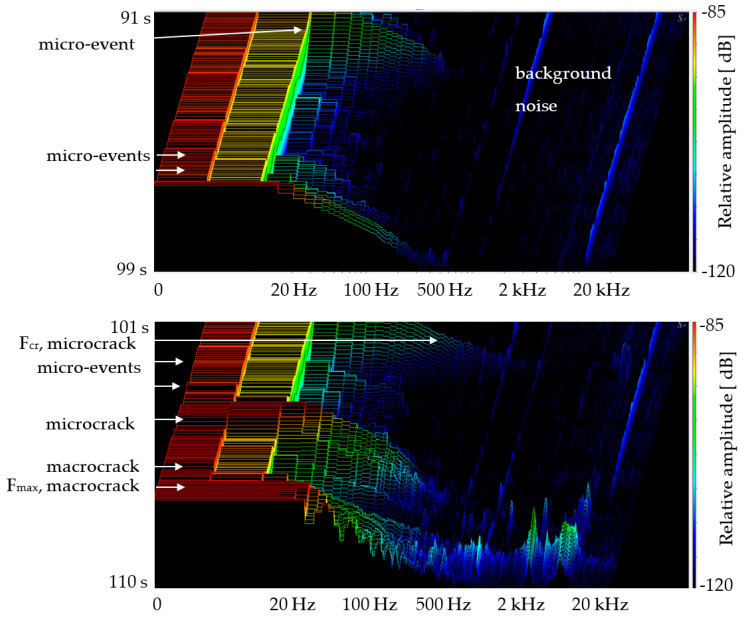
Acoustic spectrum 3D—2/M1 sample during compressive test.

**Figure 7:**
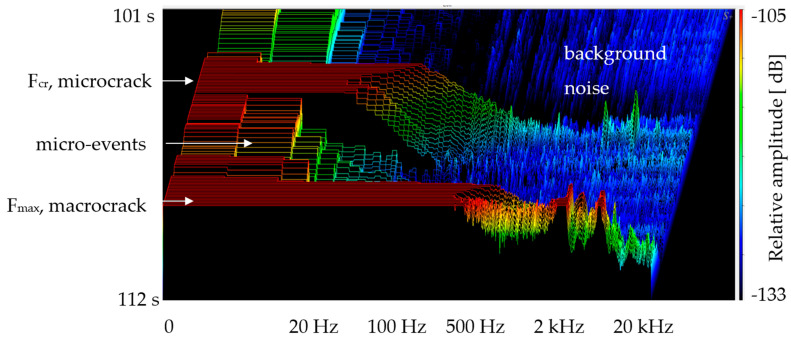
Acoustic spectrum 3D—3/M1 sample during compressive test.

The recorded acoustic effects are presented in Figure 4 (1/M1), Figure 5 (2/M2), Figure 6 (2/M1) and Figure 7 (3/M1). The 3D acoustic spectra illustrate the occurrence of micro-events, microcracks and macrocracks. Areas of concentration of acoustic effects before formation of the macrocrack at the f_max_ have been marked. The occurrence of micro-events in the infrasound range was correlated with f_cr_ in Figure 4 and Figure 6. The appearance of microcracks associated with f_cr_ is shown in Figure 5 and Figure 7. In all cases, the concentrations of micro-events and microcracks can be observed before the macrocrack forms at the f_max_.

The traverse displacement time between the f_cr_ and f_max_ (based on Table 3, Figure 3) is t_s_ = 17.0 s (1/M1), t_s_ = 17.4 s (2/M2), t_s_ = 17.0 s (2/M1) and t_s_ = 7.6 s (3/M1). The time between the first micro-event (microcrack, f_cr_) and macrocrack f_max_ based on the 3D sound spectrum is presented in Figure 4, Figure 5, Figure 6 and Figure 7 (left side).

## 4. Discussion

The results of the research confirm the possibility of correlating the processes of macro- and microcrack formation with acoustic effects before and after f_max_, Figure 4, Figure 5, Figure 6 and Figure 7. It was also confirmed that the concentration of microcracks enables the prediction of macrocrack formation at f_max_ in medium and high frequencies. Macro-destruction processes such as reinforcement fracture, pull-out or crack propagation are possible by analyzing the intensity and amplitude of acoustic signals using the 3D acoustic spectrum.

The 2/M2 microphone sample (higher-frequency range) allows the detection of macrocracks, including their concentrations, before, at and after the f_max_ point (Figure 5). However, no micro-events were detected and the identification of microcracks at f_cr_ remains unclear. The traverse displacement time between the f_cr_ and f_max_ is t_s_ = 17.4 s (Table 3, Figure 3) and the time between the first microcrack and macrocrack f_max_ is 13 s, Figure 5. It indicates that this kind of microphone (M2) does not record micro-events (microcracks), which should be found at f_cr_ approximately 17.4 s before f_max_. In the case of other samples in which the M1 microphone (recording infrasound) is used, the correlation between f_cr_ and the first microevent can be determined in Figure 4, Figure 6 and Figure 7.

**Figure 8:**
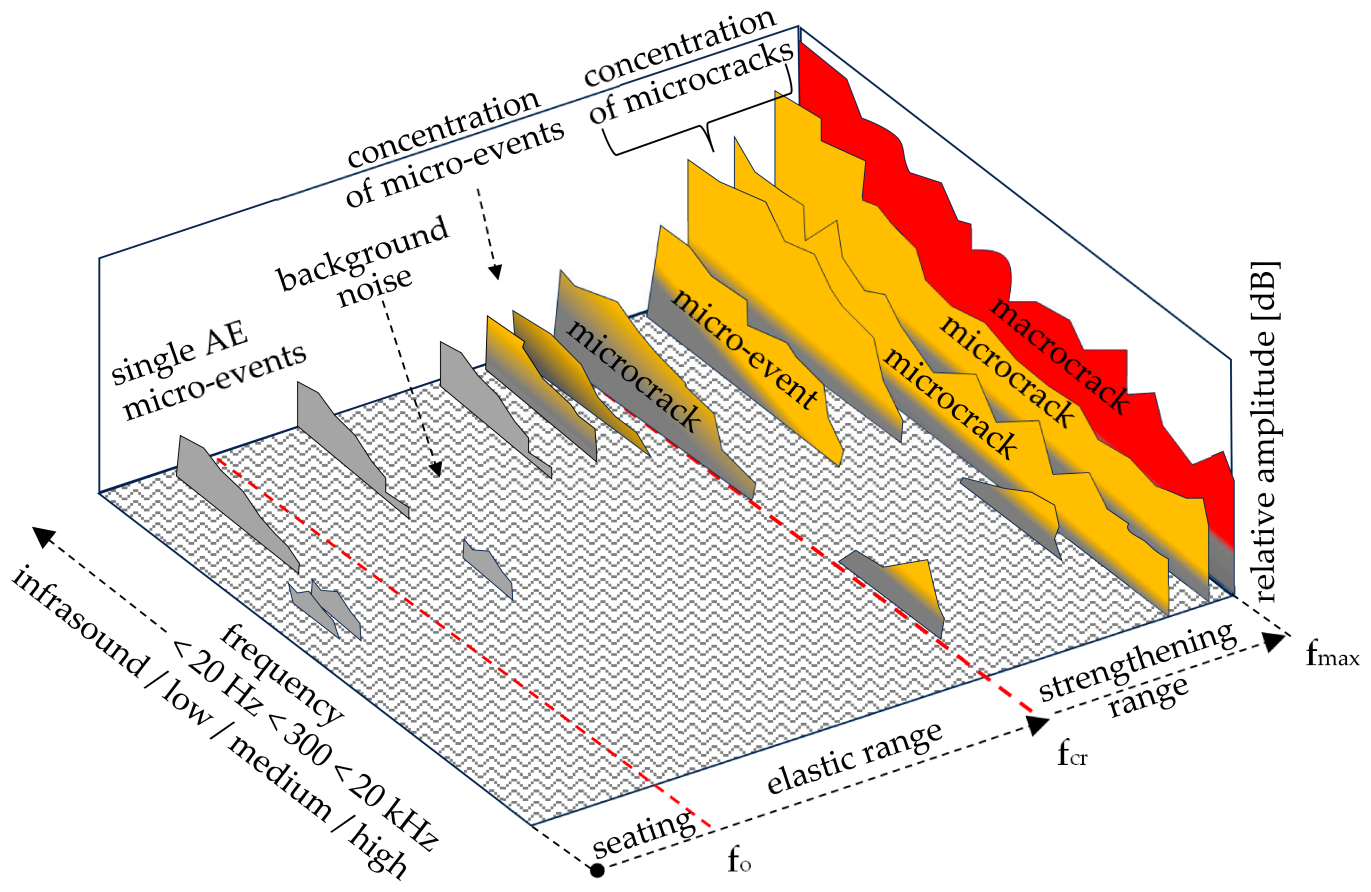
Acoustic spectrum diagram 3D—identification of f_cr_ and f_max_ in cement composites under load.

In cement composites, where the changes in the linear load–displacement correlation are not significant (resulting in low acoustic effect at f_cr_), this indicates the formation of micro-events or microcracks. This early-stage damage process makes it difficult to determine. This paper focused on the possibility of correlating 3D acoustic spectrum effects with the f_cr_ point and the strengthening range of A_S_. The scheme of the results is presented in Figure 8. It has been demonstrated that correlating acoustic events with the damage process enables the determination of f_cr_ and the prediction of macrocrack formation in cement composites at f_max_.

Previous attempts to detect acoustic effects were limited by the frequency range of the microphones and did not cover the infrasound range. A comparison of Figure 4, Figure 5 and Figure 6 shows that using the M1 microphone, which operates in the low-frequency and infrasound range, enables the detection of micro-events. Microphone measurements in the infrasound range provide a significantly better correlation between micro-events and f_cr_. In the present tests, micro-events were identified in the infrasound (<20 Hz) and in the low-frequency range.

This study did not investigate the nature of macro-events occurring in the infrasound range. Such events may include the initiation of microcracks, minor surface damage or the detachment of aggregates or reinforcement from the matrix. Determining these phenomena will require separate future studies aimed at correlating them with acoustic effects.

Macrocracks (including the f_max_ macrocrack) characterize acoustic effects in the range from infrasound (<20 Hz) to high frequencies 20 kHz. The identification of macrocracks and microcracks is possible by analyzing the amplitude and intensity of the acoustic spectrum (Figure 4, Figure 5, Figure 6 and Figure 7). The highest amplitudes and sound levels are correlated with f_max_, while the lowest correspond to micro-events and microcracks occurring with f_cr_ and the strengthening range A_S_. The general conclusions can also be extended to cement composites without fiber reinforcement, as the load–displacement curve in these materials during the compression test is similar (also characterizing f_cr_ and f_max_ points). In sample 1/M1, the amount of dispersed reinforcement (10 kg/m^3^) is relatively low, as is typical in industrial floors to reduce shrinkage. This fiber content does not significantly change the load–displacement correlation in the compression test of traditional concrete. Similar conclusions can be stated in traditional cement composites.

In evaluating the durability of both traditional and non-traditional cement composites, fmax is usually used as a reference point. The results indicate significant differences in stress and the corresponding deformations at f_cr_ and fmax. For sample 1/M1 (the lowest fiber content of 10 kg/m^3^), the difference in load F at f_cr_ and f_max_ is 19.7% and the difference between the displacements d_cr_ and d_max_ is 6.7%. Furthermore, this sample exhibits the highest deformability in the strengthening range As and the greatest number of recorded acoustic events correlated with the destruction process, including the absorbed energy W_s_. These results indicate that, for assessing the durability of cement composites with a significant strengthening range A_s_, the stress corresponding to f_cr_ and not to f_max_ should be considered.

Presented samples confirmed the possibility of identifying micro-events in infrasound 3D spectrum and the lack of such a possibility in the range of medium and high frequencies. The possibility of observing the presented correlation was found each time in cement composite tests (with or without dispersed reinforcement). This conclusion refers mainly to our own studies included in the references and does not exclude the possibility of identifying micro-events by other methods in a different frequency range [41,42,43,44,46,47]. The paper indicates the possibility of designating micro-events and further quantitative research is needed to identify this micro-destruction process. An analysis of micro-events using SEM, optical microscopy, X-ray tomography, DIC, and ultrasound imaging is essential. This recognition is important due to the possible significant impact of micro-events on materials durability.

## 5. Conclusions

The concentration of micro-events and microcracks in cement composites with the 3D acoustic spectrum in the infrasound range (at low acoustic intensities) enables the determination of the critical point f_cr_. It was indicated that it is difficult to determine microdefects in the medium- and high-frequency range.

It was stated that further research should correlate micro-events with the 3D acoustic spectrum in cement composites using the infrasound (such as reinforcement or aggregate detachment, microcrack initiation, etc.).

The assessment of the damage process in cement composites requires correlation with 3D acoustic spectra over a wide frequency range from infrasound (associated with micro-events at f_cr_) to medium- and high-frequency signals related to macrocracks and reinforcement destruction. The concentration of acoustic effects indicates the initiation and propagation of micro and macrocracks at f_cr_ and f_max_.

Significant differences in strength and displacement at f_cr_ and f_max_ during the compressive test of cement composites and a large amount of absorbed energy (micro-destruction process in the strengthening range) were demonstrated. These results indicate that for an accurate assessment of the durability of cement composites, the stress and the displacement corresponding to f_cr_ should be considered and determined by 3D acoustic spectra in the range of infrasound.

## Figures and Tables

**Table 1:** Microphones used for acoustic measurements.

Microphone	Sensitivity	Dynamic Range	Frequency Range
M1–Gras 46AZ	50 mV/pa	17 dB(A)–138 dB re20 mPa	0.5 Hz–20 kHz
M2–Gras 46BE	3.6 mV/pa	35 dB(A)–160 dB re20 mPa	4 Hz–70 kHz

**Table 2:** Tested samples.

Symbol	Microphone	Fiber Volume
1/M1	M1	10 kg/m^3^
2/M1	M1	15 kg/m^3^
2/M2	M2	15 kg/m^3^
3/M1	M1	17 kg/m^3^

**Table 3:** The mechanical properties of concrete in the compressive strength test.

Specimen	F_cr_ [kN]	d_cr_ [mm]	f_cr_ [MPa]	F_max_ [kN]	d_max_ [mm]	f_max_ [MPa]	tgα [kN/mm]	d_S_ [mm]	W_S_ [J]	t_S_ [s]
1/M1	1001	1.963	44.5	1192	2.975	53.0	510	1.012	1256	17.0
2/M1	1165	1.649	51.8	1362	2.321	60.5	706	0.672	965	17.4
2/M2	1203	1.675	53.5	1395	2.212	62.0	718	0.537	799	17.0
3/M1	1275	1.572	56.7	1361	1.881	60.5	811	0.309	471	7.6

## Data Availability

The original contributions presented in this study are included in the article. Further inquiries can be directed to the corresponding author.
